# Outcome of visuospatial dysfunction assessment in patients with Parkinson’s disease using mobile application software

**DOI:** 10.3389/fnagi.2023.1108166

**Published:** 2023-02-23

**Authors:** Xu Shao, Kang Wang, Yulian Zhang, Xueke Zhen, Fen Dong, Hong Tian, Yanbing Yu

**Affiliations:** ^1^Department of Neurosurgery, China-Japan Friendship Hospital, Beijing, China; ^2^Department of Neurology, China-Japan Friendship Hospital, Beijing, China; ^3^Department of Clinical Research and Data Management, Center of Respiratory Medicine, China-Japan Friendship Hospital, Beijing, China; ^4^Institute of Respiratory Medicine, Chinese Academy of Medical Sciences, Beijing, China; ^5^National Clinical Research Center for Respiratory Diseases, Beijing, China

**Keywords:** Parkinson’s disease, cognitive impairment, visuospatial dysfunction, mobile application, clock drawing test, cube copying test

## Abstract

**Background:**

Visuospatial dysfunction and cognitive impairment are common in Parkinson’s disease (PD), which draw increasing attention in the current literature. But clinicians still lack rapid, effective and unified cognitive battery for visuospatial assessment.

**Objective:**

A new approach was studied to explore the feasibility of using mobile application software (APP) to evaluate visuospatial dysfunction in patients with PD and compared with traditional assessment tools. We aimed to verify the threshold score of the APP for early diagnosis.

**Materials and methods:**

A total of 41 patients with PD underwent assessments using several test modules including Digit Symbol Test (DST), Visual Organization Test (VOT), Facial Recognition Test (FRT), Vocabulary Memory Test (VMT) of this APP, as well as Clock Drawing Test (CDT), Cube Copying Test (CCT) and the Mini-Mental State Examination (MMSE) for comparison. Among the 41 PD patients, 30 individuals were found to have visuospatial dysfunction based on CDT score < 5 and CCT score of<18 while the remaining 11 patients served as control.

**Results:**

There were statistically significant differences in DST, VOT, and FRT scores (all *p* ≤ 0.001 for group comparisons). DST, VOT, and FRT-1 were significantly correlated with MMSE, CDT and CCT and the correlations were moderate or fairly strong. For visuospatial dysfunction diagnosis, all the areas under curves (AUC) of DST, VOT, and FRT-1 were statistically significant (*p* < 0.0001, *p* = 0.0002, and *p* = 0.0002, respectively). The estimates and 95% confidence intervals of AUC were 0.8303 (0.6868, 0.9739), 0.8045 (0.6423, 0.9668), and 0.7833 (0.6344, 0.9322), respectively. Their cut-off points for visuospatial dysfunction were 26, 17, and 19, respectively. After dichotomization by the cut-off points, DST had high sensitivity of 96.67% while VOT and FRT-1 had high specificity of 81.82 and 90.91%.

**Conclusion:**

This study demonstrated that visuospatial disorders was highly prevalent in PD patients, and the APP used in study could be a practical clinical screening tool for visuospatial ability assessment with high sensitivity and specificity.

## 1. Introduction

Parkinson’s disease is a multisystem neurodegenerative disease with motor symptoms characterized by resting tremor, bradykinesia, muscle rigidity, and postural gait abnormality (Yang et al., 2016). In addition to motor impairments of patients with PD present, non-motor impairments manifest a variety of neuropsychiatric symptoms mainly including sleep, behavior and cognition. The impairments in cognitive functions, such as memory, executive function, visuospatial skills and language in PD, are drawing increasing attention in the current literature (Aarsland et al., 2021). Non-motor symptoms are predictive of decreased ability to perform daily living, especially visuospatial impairment, which is distinguished by its early appearance, divided into visuospatial functions impairment and visuospatial cognition impairment. Previous cross-sectional studies have shown that PD patients may have deficits in executive functioning, concentration, facial recognition, recent and working memory (Tachibana, 2013; Galtier et al., 2014). Various neuropsychological tests are available for diagnosing visuospatial impairment, however, motor symptoms including tremor and muscle rigidity can be challenging for the diagnostic procedure in PD patients. CDT, CCT, and MMSE are traditional screening instruments for dementia as a measure of visuospatial dysfunction, but requires fine motor ability. In order to reduce the bias of motor factors, mobile apps were developed as a screening tool for cognition impairment to investigate the characteristics, distribution and possible related factors of visuospatial impairment in PD patients.

## 2. Materials and methods

### 2.1. Study design and population

We consecutively enrolled patients who visited our study group for PD between November 2021 and September 2022. Eligible patients were those who were diagnosed with PD according to the International Parkinson and Movement Disorder Society (MDS) criteria (Postuma et al., 2015). Exclusion criteria were any neurological disorder other than PD including parkinsonism secondary to trauma or drugs, metabolic diseases, encephalitis, progressive supranuclear palsy, essential tremor, and hepatolenticular degeneration. All eligible patients underwent assessment *via* APP tests including DST, VOT, FRT, and VMT in the APP with raw scores recorded, at the same time, CDT, CCT, and MMSE were also evaluated as classic evaluation tools for comparison. Patients with the CDT score of 5 (Spenciere et al., 2017) and the CCT score ≥ 18 (Bu et al., 2013) were classified to no visuospatial disorder group, while patients with the CDT score < 5 or CCT score < 18 were classified to visuospatial disorder group. Information on patients’ demographic characteristics and clinical profile were collected from medical records. This study was performed in accordance with the Declaration of Helsinki and approved by the ethics committee of China-Japan friendship Hospital (2020-129-K82). All participants gave their informed consent to participate in the study in written form.

### 2.2. Neuropsychological assessment

A cognitive assessment application developed by Dr. Xiaodong Pan, Department of Neurology, Fujian Medical University Union Hospital was used for tests, including DST, VOT, FRT, and VMT. These tests could assess visual acuity, visual speed of processing and attention, visual and verbal memory, visual constructional abilities and executive functions. The assessment application could be downloaded and used through the Android App Market and Apple App Market for free.

#### 2.2.1. Digit symbol test

Digit symbol test (DST) was conducted to assess visual processing speed, visual shape judgment and motor coordination through the association of numbers and symbols. Numbers 1–9 each correspond to a symbol. The participant was required to select the symbol matching the number on the screen as soon as possible within 1 min according to the given list of numbers and symbols. The software automatically scored according to the number of correct selections and the full score was 54.

#### 2.2.2. Visual organization test

Visual organization test (VOT) was conducted to assess visual constructional ability and mental rotation function. In this test, a complete object picture was divided into several parts by image segmentation and rotation. The participant was asked to identify local features or abstract combinations, and then select the appropriate answer. The software automatically scored based on the number of correct selections and the full score was 30.

#### 2.2.3. Facial recognition test

Facial recognition test (FRT) was conducted to assess facial recognition and visual perception. In this study, the spatial structure cognitive ability and emotional perception experience ability of facial features as the main characteristics were evaluated through the recognition of facial expressions. The first stage (FRT-1) was to choose the appropriate expression option according to the photo of face, a total of 24 questions meaning full score was 24. In the second stage (FRT-2), according to the given emoticon instruction, two corresponding facial photos were selected from eight similar pictures, a total of 16 questions meaning full score was 16. The software automatically scored based on the number of correct selections.

#### 2.2.4. Vocabulary memory test

Vocabulary memory test (VMT) was conducted to evaluate the ability of visual and verbal memory by memorizing a limited vocabulary through rapid browsing. The participant would read 12 words in 24 s in turn, each word appearing once on the screen, and then confirm which words have been read before in 24 words including 12 new words and 12 remembered words. The software automatically scored based on the number of correct answers and full score was 24.

#### 2.2.5. Clock drawing test

Clock drawing test (CDT) which had more than one version with different scoring methods seemed to be impacted quite early in the decline process of cognitive in PD. The 5-item score Shulman system was considered as an accurate method for the general use in the diagnosis of PD, requiring substantial understanding of its scoring system (Park et al., 2018). The participant was required to draw a digital clock on a white paper, with the clock indicating “10 min past 11 h” with no concern about the speed. The Shulman system indicated that a score of 5 for a perfect clock, a score of 4 for a slight visuospatial error, a score of 3 for an inaccurate representation of 10 min past 11 h when the visuospatial organization was well done, a score of 2 for moderate visuospatial disorganization of numbers such that accurate denotation of 10 min past 11 h was impossible, a score of 1 for a severe visuospatial disorganization, and a score of 0 for no reasonable representation of the clock could be made (Shulman, 2000).

#### 2.2.6. Cube copying test

The evaluation of the Cube copying test (CCT) was based on the cube assessment of Maeshima (Maeshima et al., 2004). In this test, the connections and lines in the cube were evaluated. A connection point was defined as a point where 3 lines intersect to form a vertex. Lines less than 3 mm from this point were considered accurate. Because a cube consists of 8 connections and 12 parallel lines, patients could get up to 20 points (8 + 12). The cube-copying task, which mainly measured visuospatial ability (Palmqvist et al., 2008) and motor dysfunction (Bu et al., 2013), had been shown to be deteriorated in PD.

#### 2.2.7. Mini-mental state examination

As a widely known cognitive assessment tool, MMSE was used to assess the cognitive status of people at high risk of dementia, such as AD and PD patients. But MMSE had been criticized for its lack of sensitivity, especially in mild cases of PD (Snyder et al., 2021). A normative study of Chinese elderly population showed that the optimal cut-off points for dementia screening were 16/17 for illiterate (sensitivity 87.6% and specificity 80.8%), 19/20 for individuals with 1–6 years of education (sensitivity 93.6% and specificity 92.7%), and 23/24 for individuals with 7 or more years of education (sensitivity 94.3% and specificity 94.3%; Li et al., 2016).

### 2.3. Statistical analysis

The statistical analyses of the patients were summarized in the tables and figures to provide detailed information. Data were represented as number (percentage) for categorical variables and mean ± SD for continuous variables where appropriate. To compare differences in demographic information, clinical profile, and visuospatial assessment scores, two independent *T*-test were conducted for normally distributed continuous variables while non-parametric Wilcoxon rank sum test was used for non-normally distributed ones. Chi-square or Fisher’ exact test was utilized for categorical variables. Correlations between APP assessments and MMSE, CCT, and CDT were quantified using Pearson or Spearman correlation coefficients based on distribution of variables. Areas under the curve (AUC) of moderately or strongly correlated APP assessments for visuospatial disorder were tested and their cut-off points with optimal Youden index were determined. Sensitivity, specificity, positive and negative prediction were estimated. All statistical analyses were performed by using SAS V9.4 (SAS Institute, Cary, North Carolina, United States).

## 3. Results

### 3.1. Demographic data and cognitive evaluations

The demographic data, clinical profiles and cognitive assessment outcomes of the 41 patients were summarized in Table 1. Among the study patients, 30 individuals were grouped as visuospatial disorder while the resting 11 patients were classified as non-visuospatial disorder group. Age, gender, and education level did not differ statistically between the two groups. However, there were significant differences in the scores of classic assessments and APP assessments such as DST, VOT and FRT. Group comparison of VMT scores did not reveal significant difference, indicating that visuospatial dysfunction in PD patients was not accompanied by word transient memory disorders.

**Table 1:** Demographic, clinical, and visuospatial profile of patients with PD.

Variable^a^	Value	All *n* = 41	Visuospatial disorder group *n* = 30	None visuospatial disorder group *n* = 11	*p* value
Demographic characteristics					
Age		62.0 ± 9.5	63.3 ± 8.5	58.2 ± 11.2	0.1239
Gender	Male	20 (48.78%)	13 (43.33%)	7 (63.64%)	0.2492
	Female	21 (51.22%)	17 (56.67%)	4 (36.36%)	
Education level	College or higher	7 (17.07%)	4 (13.33%)	3 (27.27%)	0.3608^b^
	High school or lower	34 (82.93%)	26 (86.67%)	8 (72.73%)	
Clinical profile					
Disease duration		7.0 ± 5.1	7.7 ± 5.4	5.0 ± 3.4	0.1197
Side of disease onset	Bilateral	7 (17.07%)	4 (13.33%)	3 (27.27%)	0.3681^b^
	Right	18 (43.90%)	15 (50.00%)	3 (27.27%)	
	Left	16 (39.02%)	11 (36.67%)	5 (45.45%)	
Hoehn-Yahr grade	1	4 (9.76%)	1 (3.33%)	3 (27.27%)	0.0466^b^
	1.5	2 (4.88%)	2 (6.67%)	0 (0.00%)	
	2	8 (19.51%)	4 (13.33%)	4 (36.36%)	
	2.5	7 (17.07%)	7 (23.33%)	0 (0.00%)	
	3	14 (34.15%)	10 (33.33%)	4 (36.36%)	
	3.5	1 (2.44%)	1 (3.33%)	0 (0.00%)	
	4	5 (12.20%)	5 (16.67%)	0 (0.00%)	
Visuospatial disorder assessment using APP					
Digit Symbol (DST)		19.5 ± 9.2	16.3 ± 6.7	28.3 ± 9.7	<0.0001
Visual Organization (VOT)		17.0 ± 5.2	15.5 ± 4.6	21.0 ± 4.9	0.0019
Facial Recognition-1 (FRT-1)		18.9 ± 3.5	18.1 ± 3.6	21.1 ± 1.9	0.0058
Facial Recognition-2 (FRT-2)		9.6 ± 3.1	8.9 ± 3.0	11.5 ± 2.4	0.0164
Vocabulary Memory (VMT)		16.7 ± 4.2	16.2 ± 4.5	18.1 ± 2.8	0.3130
Visuospatial disorder assessment by classic tools					
MMSE		23.9 ± 4.1	22.6 ± 4.1	27.4 ± 1.3	<0.0001
CCT		14.1 ± 4.0	12.3 ± 3.1	19.1 ± 0.8	<0.0001
CDT	1	3 (7.32%)	3 (10.00%)	0 (0.00%)	<0.0001^b^
	2	6 (14.63%)	6 (20.00%)	0 (0.00%)	
	3	16 (39.02%)	16 (53.33%)	0 (0.00%)	
	4	5 (12.20%)	5 (16.67%)	0 (0.00%)	
	5	11 (26.83%)	0 (0.00%)	11 (100.0%)	

^a^Continuous variables were expressed as mean ± SD. Categorical variables were expressed as number (percentage). ^b^Fisher exact test.

### 3.2. Exploratory correlation analysis between APP assessments and classic tests scores

**Figure 1:**
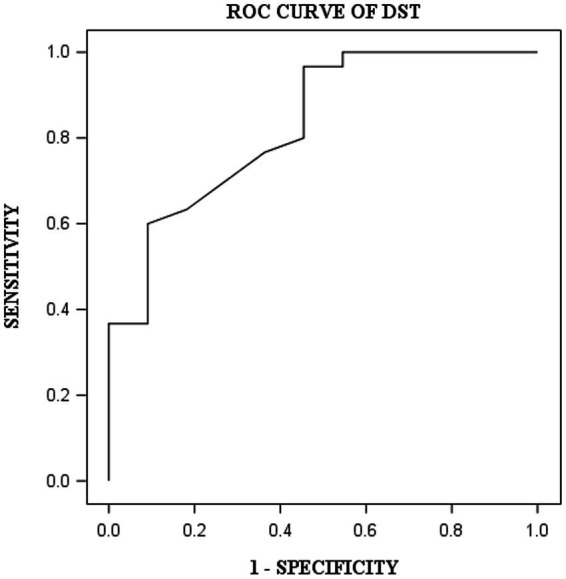
ROC curve of DST for visuospatial disorder.

**Figure 2:**
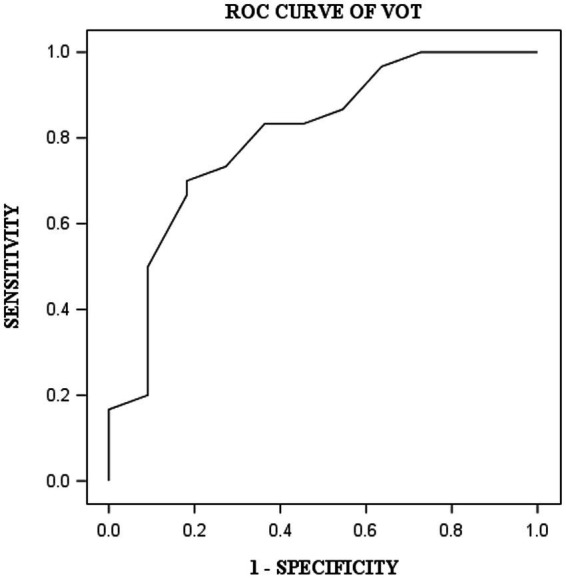
ROC curve of VOT for visuospatial disorder.

**Figure 3:**
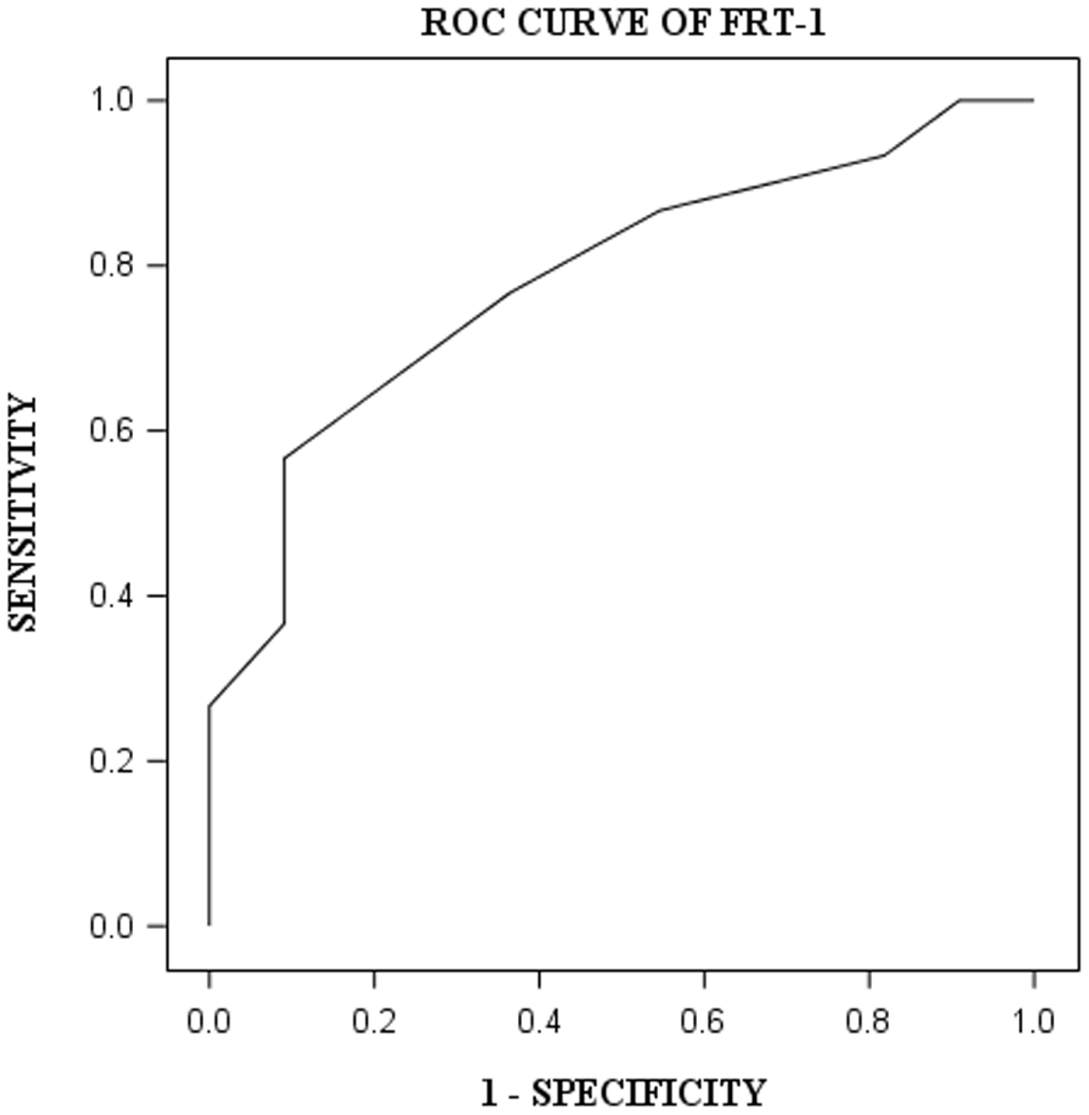
ROC curve of FRT-1 for visuospatial disorder.

DST, VOT, and FRT-1 were significantly correlated with MMSE, CCT and CDT with strong or moderate correlations (Table 2). The correlation coefficients of FRT-1 with MMSE, CCT and CDT were consistently higher than those of FRT-2 with those classic indicators, particularly with CDT. Thus, we analyzed FRT-1 in the following ROC analysis instead of FRT-2. Figures 1–3 illustrated ROC curves of the three metrics for visuospatial disorder diagnosis. All the AUCs of DST, VOT, and FRT-1 were significantly different from 0.5 (*p* < 0.0001, *p* = 0.0002, and *p* = 0.0002, respectively). Their estimates and 95% confidence intervals were 0.8303 (0.6868, 0.9739), 0.8045 (0.6423, 0.9668), and 0.7833 (0.6344, 0.9322) respectively. Comparison of AUCs between DST and FRT-1 demonstrated insignificant differences (*p* = 0.5026) and AUC of VOT did not differ from that of FRT-1, neither (*p* = 0.8251). The cut-off points of DST, VOT, and FRT-1 were 26, 17 and 19, respectively. Based on the thresholds (DST ≤ 26, VOT ≤ 17, FRT-1 ≤ 19), DST had high sensitivity with 0.9667 (0.8278, 0.9992) while VOT and FRT-1 had excellent specificity with 0.8182 (0.4822, 0.9772) and 0.9091 (0.5872, 0.9977). DST had both high positive and negative predictions while the latter two metrics had high positive prediction. It suggested the combination of APP assessments could obtain objective visuospatial ability assessment quantitative scores (Table 3).

**Table 2:** Correlation analysis between APP assessments and classic tests scores.

APP assessments		Classic tools		
		MMSE	CCT	CDT
DST	Correlation	0.31807	0.56972^a^	0.56558
	*p* value	0.0427	0.0001	0.0001
VOT	Correlation	0.70403	0.59044^a^	0.58703
	*p* value	<0.0001	<0.0001	<0.0001
FRT-1	Correlation	0.54962	0.43964	0.55016
	*p* value	0.0002	0.004	0.0002
FRT-2	Correlation	0.51772	0.42436^a^	0.44164
	*p* value	0.0005	0.0057	0.0038
VMT	Correlation	0.42423	0.22092	0.29506
	*p* value	0.0057	0.1651	0.0611

^a^Pearson correlations between CCT and DST, VOT, FRT-2 were analyzed, respectively given the normally distributed variables while other correlations were spearman correlations due to ordinal variables or non-normal distributions of continuous variables.

**Table 3:** Sensitivity, specificity, and predictions of DST, VOT, and FRT-1 for visuospatial disorder.

Variable	Estimate (95% confidence interval^*^)			
	Sensitivity	Specificity	Positive prediction	Negative prediction
DST ≤26	0.9667 (0.8278, 0.9992)	0.5455 (0.2338, 0.8325)	0.8529 (0.6894, 0.9505)	0.8571 (0.4213, 0.9964)
VOT ≤17	0.7000 (0.5060, 0.8527)	0.8182 (0.4822, 0.9772)	0.9130 (0.7196, 0.9893)	0.5000 (0.2602, 0.7398)
FRT-1 ≤ 19	0.5667 (0.3743, 0.7454)	0.9091 (0.5872, 0.9977)	0.9444 (0.7271, 0.9986)	0.4348 (0.2319, 0.6551)

^*^Exact binomial confidence limits.

## 4. Discussion

Although the mechanism of visuospatial dysfunction in PD patients remains unclear, relevant research results show that visuospatial processing division include dorsal and ventral streams. The dorsal stream starts from the occipital lobe and projects to the parietal lobe, called the occipitoparietal pathway, which is related to the spatial location of objects, and its structure includes bilateral superior parietal cortex and lateral occipital lobe. The ventral stream is the occipitotemporal pathway, which is related to face recognition. These structures include the middle occipital gyrus, the occipitotemporal junction area, the parahippocampal gyrus, etc. The two pathways send fibers directly or indirectly to the prefrontal cortex through the ventroposteromedial thalamic nucleus and the corticospinal tract. The prefrontal cortex plays a role in keeping spatial information updated in real time in visuospatial function (Kravitz et al., 2011).

Recently, symptoms about visuospatial dysfunction in PD patients, such as stumble and becoming lost, have attracted attention as serious social problems. Visuospatial function is impaired from the early phase (Zhang et al., 2020). It is known that the focus of route finding is associated with the striatal dopamine depletion, dopamine transporter availability in the caudate, anterior putamen, and ventral striatum was directly associated with attention/working memory, frontal/executive, and visuospatial functions (Chung et al., 2018). Berlot et al. (2022) evaluated relationships between structure of the cholinergic basal forebrain, medial temporal lobe and cognition by measuring volumes of the cholinergic basal forebrain nuclei, the entorhinal cortex, the hippocampus and its subfields in PD patients and controls. Their data implied that the integrity of the cholinergic basal forebrain was associated with subregional hippocampal volume, and influencing visuospatial function.

It is difficult to find a common cognitive assessment battery across studies. In addition, some instruments traditionally used in PD may not be adequate for use in visuospatial function assessment. More than 53 assessment tools can be used in Parkinson’s cognitive impairment including the Montreal Cognitive Assessment (MoCA), the Digit Span, the Trail Making Test, the Semantic Fluency test, the Rey Auditory Verbal Learning Test, the Brief Visuospatial Memory Test-Revised, the Boston Naming Test and the CDT, etc. (SeverianoE Sousa et al., 2022). MMSE and MoCA which including CCT and CDT are practical and efficient screening tools for PD dementia with visuospatial dysfunction (Ohta et al., 2014). In addition, the Pentagon Copying Test, Judgment of Line Orientation Test, Visual Form Discrimination Test, Facial Recognition Test, Symbol Digit Modalities Test can be selected for visuospatial tests (Garcia-Diaz et al., 2018). As classic assessment tools, CDT and CCT are widely used in the assessment of PD patients because they are easy to understand and requiring less time (Scarpina et al., 2020; Mori et al., 2021; Srivastava et al., 2022). However, their limitations and shortcomings are also obvious. CDT and CCT require complex and delicate movements, which means more difficult to perform for PD patients. It is perplexing to judge whether their test results imply the decline of visuospatial ability or the difficulty of execution caused by motor dysfunction. Some clinicians even think that the visuospatial disorder may not exist if the factors of motor ability decline are removed (Brown and Marsden, 1986). Therefore, the selection of assessment tools with high sensitivity and good specificity can get more objective results and improve the efficiency of clinical work. The APP assessments used in our study could be applied in mobile devices such as mobile phones or tablets, which can facilitate the assessment by clinicians, requiring a smaller range of limb movement and a lower level of delicate movement. For patients, the difficulty of completing tests is significantly reduced by using their fingers to click on the screen compared with drawing clock and copying cube. In the meantime, it has excellent sensitivity and specificity for visuospatial function test.

Cognitive function is affected by many factors. The present study had shown that general cognitive function, executive function, memory, and information processing speed in PD patients were related to educational level, while no significant association was showed between educational level and visuospatial function, language in PD patients (Gu and Xu, 2022). At present, most of the commonly used neuropsychological assessment tools were developed in Anglosphere cultures. Statucka and Cohn (2019) studied the cognitive function differences between Canadian immigrants and aborigines after diagnosis of PD, and found that the immigrant group showed lower scores and greater rates of deficits on all visuospatial and some executive function tasks, but not on attention or memory measures. These biases could not be explained by demographic and clinical variables as groups were comparable. Because the differences between groups were strongly mediated by the Historical Index of Human Development of the participant’s country of birth, which reflects economic, health, and educational potential of a country at the time of birth. The assessment APP used in our study took full account of the cultural differences between China and the West, with less internal correlation on factors such as educational level, cultural differences, ethnic habits, and economic status. The requirements of the APP were simple and feasible for the PD patient to complete, and allowed clinicians to achieve more objective and consistent neurophysiological assessments. Because the evaluation criteria of APP were invariable, which was different from MMSE, CDT and CCT, there had no interference from subjective factors of clinicians.

At present, the treatment of visuospatial disorders is mainly cognitive rehabilitation training, which can be combined with transcranial direct current stimulation (tDCS) and transcranial magnetic stimulation (TMS) (Veldema et al., 2020), as well as pharmacological treatment and DBS surgery. Pharmacotherapy include dopaminergic and cholinergic treatment. Related studies have shown that there are different outcomes in the effect of dopaminergic treatment, and no unified conclusion can be obtained from those studies. Cholinergic treatment may be the best option for future research (van der Kemp et al., 2017). Because the existing literature is very sparse and studies have various methodological limitations, it is currently not possible to either support or reject the effects of DBS surgery on cognitive function. Some studies reported that DBS might lead to the decline in visual constructive and visuospatial skills, while other articles shown no significant change pre and post-operatively (BarbozaE Barbosa and Fichman, 2019).

### 4.1. Limitations

Three limitations of this study must be considered. First, during the COVID-19 pandemic, the government recommended reducing unnecessary social activities, and people preferred reduce the frequency of visits to the hospital. As a result, it was difficult to recruit participants, and the sample size was small. PD patients in this study were from one medical center and the findings may not reflect the state of PD general population. Second, the details of the pharmacotherapy history of participants were not recorded and the possible intrinsic correlation between the timing of the test and the patient’s medication schedule was imperceptible. Third, the incidence of visuospatial disorders might vary according to the different neuropsychological assessments used, and the most suitable tool for estimating visuospatial disorders in PD is still a matter of controversy. CDT, CCT and MMSE were chosen as traditional assessment tools, but they may still have some limitations and may lead to bias in the results. In the future, we need more research about consistency of assessment tools in particular and longitudinal studies about possible risk factor associated with visuospatial dysfunction.

## 5. Conclusion

The results of this study showed that the incidence of visuospatial disorders in PD patients was high, and there was still a lack of rapid, effective and unified cognitive assessment battery. Assessments in APP had higher sensitivity and better specificity, which could help clinicians to diagnose PD patients with visuospatial disorders simply and quickly, and we also explored threshold score for diagnosing visuospatial disorders through this APP. These findings could also improve early rehabilitation guidance and pharmacological interventions.

## Data availability statement

The raw data supporting the conclusions of this article will be made available by the authors, without undue reservation.

## Ethics statement

The studies involving human participants were reviewed and approved by Medical Ethics Committee of China-Japan friendship Hospital (2020-129-K82). The patients/participants provided their written informed consent to participate in this study.

## Author contributions

XS, HT, and KW contributed to conception and design of the study. YZ and XZ contributed to the data extraction and organized the database. XS and FD contributed to methodology and performed the statistical analysis. HT and YY contributed to supervision, funding acquisition and project administration. All authors contributed to the article and approved the submitted version.

## Funding

This study was funded by the Capital’s Funds for Health Improvement and Research (CFH, grant number 2020-2-4061).

## Conflict of interest

The authors declare that the research was conducted in the absence of any commercial or financial relationships that could be construed as a potential conflict of interest.

## Publisher’s note

All claims expressed in this article are solely those of the authors and do not necessarily represent those of their affiliated organizations, or those of the publisher, the editors and the reviewers. Any product that may be evaluated in this article, or claim that may be made by its manufacturer, is not guaranteed or endorsed by the publisher.
