# Supplementary material for: Outcome of visuospatial dysfunction assessment in patients with Parkinson’s disease using mobile application software
Source: Front Aging Neurosci. 2023 Feb 23;15:1108166. doi: 10.3389/fnagi.2023.1108166 (PMC9996065; doi:10.3389/fnagi.2023.1108166)
Supplement: Supplementary file 1 [file Data_Sheet_1.docx]

Supplementary Material

# Supplementary Data


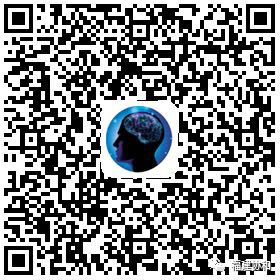


**Scan picture to download APP in this study for IOS system.**

**Install software for Android system is available by the authors.**

# Supplementary Figures and Tables

**Scatter diagram in this study, but not in the manuscript.**


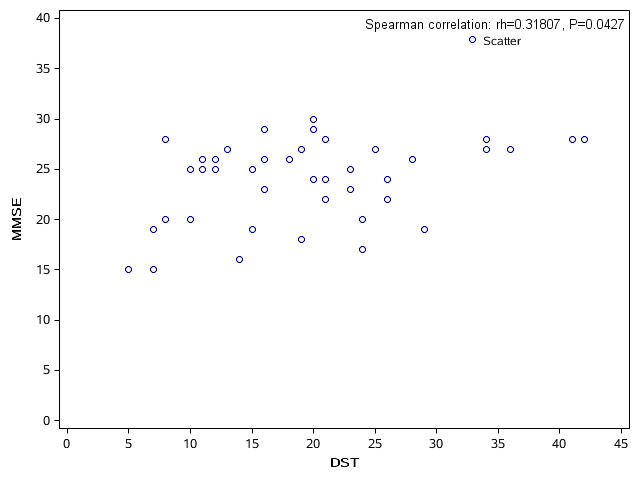
 **Supplementary Figure 1.** Scatter diagram between MMSE and DST


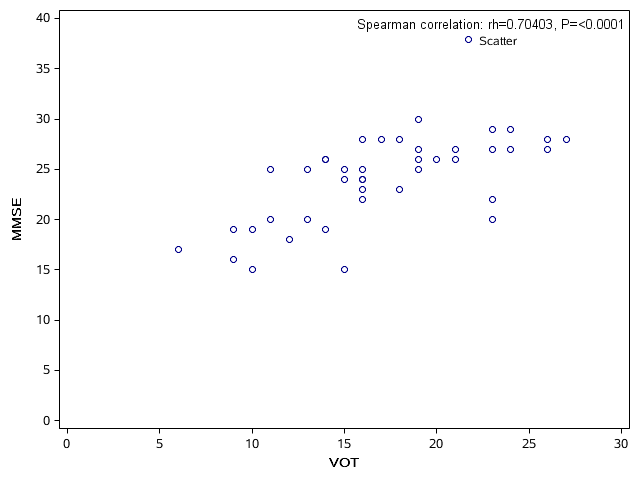


**Supplementary Figure 2.** Scatter diagram between MMSE and VOT


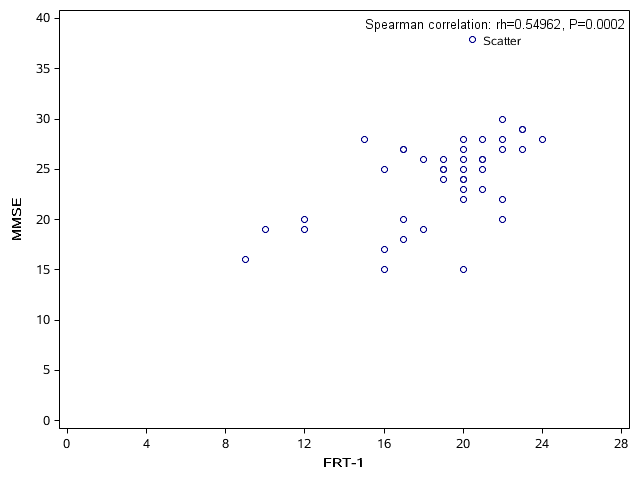


**Supplementary Figure 3.** Scatter diagram between MMSE and FRT-1


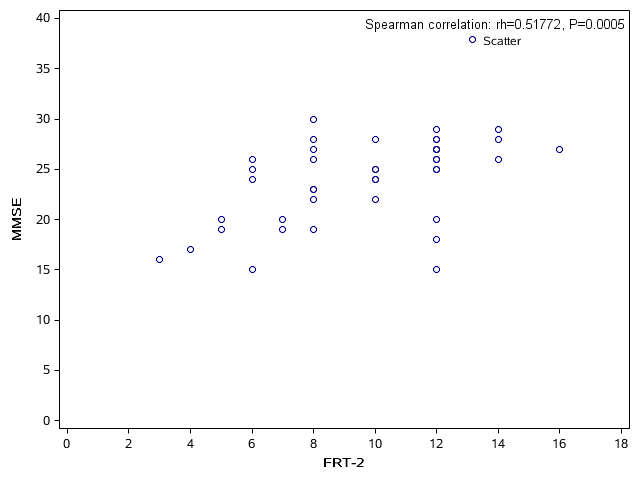


**Supplementary Figure 4.** Scatter diagram between MMSE and FRT-2


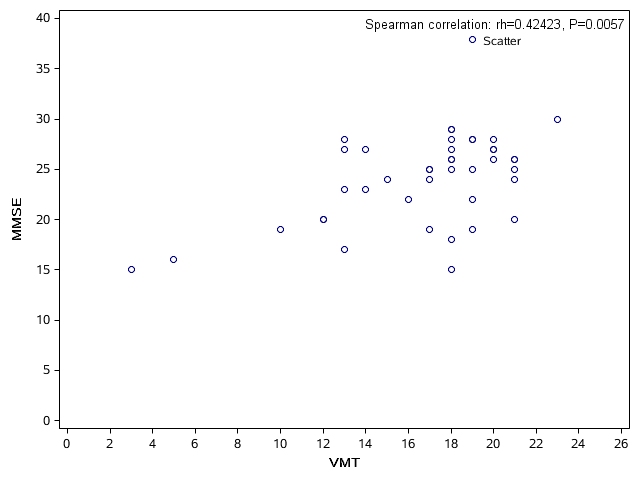
 **Supplementary Figure 5.** Scatter diagram between MMSE and VMT


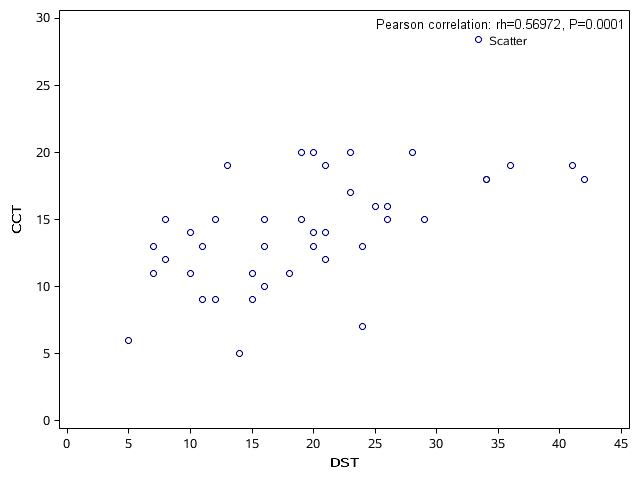


**Supplementary Figure 6.** Scatter diagram between CCT and DST


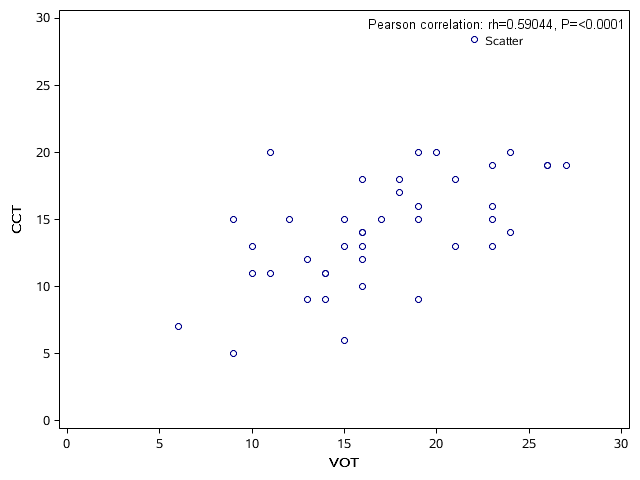


**Supplementary Figure 7.** Scatter diagram between CCT and VOT


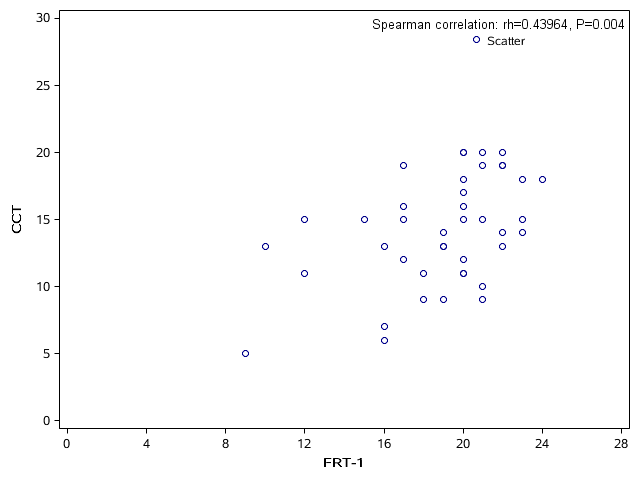


**Supplementary Figure 8.** Scatter diagram between CCT and FRT-1


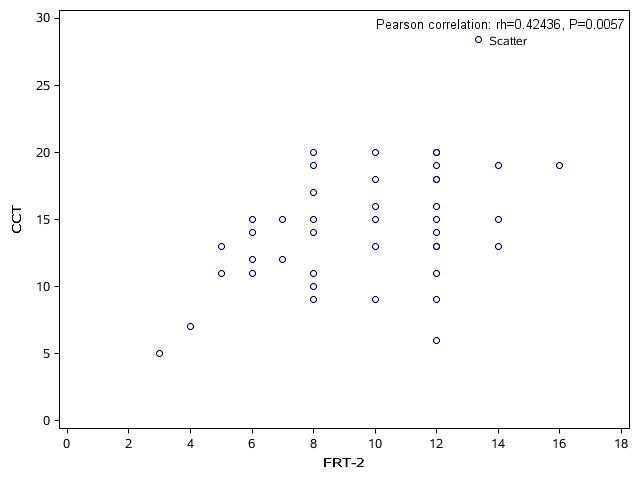
**Supplementary Figure 9.** Scatter diagram between CCT and FRT-2


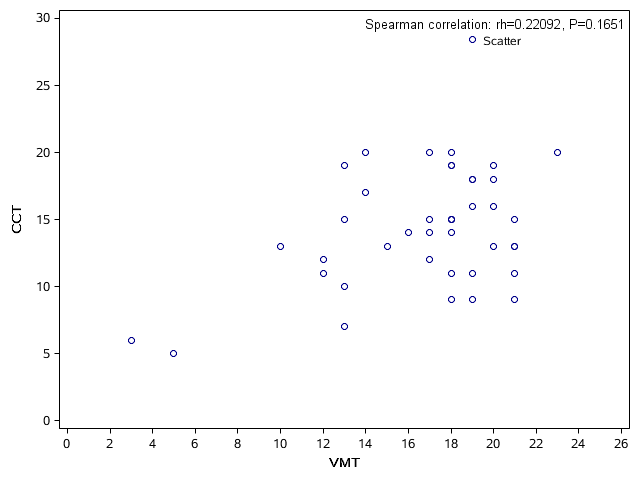
**Supplementary Figure 10.** Scatter diagram between CCT and VMT


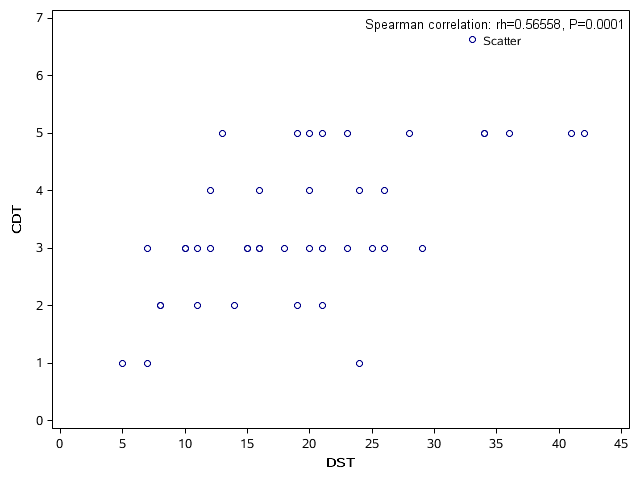
 **Supplementary Figure 11.** Scatter diagram between CDT and DST


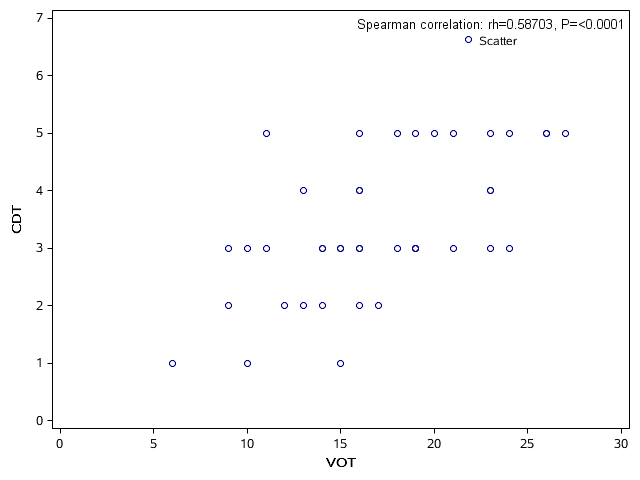
 **Supplementary Figure 12.** Scatter diagram between CDT and VOT


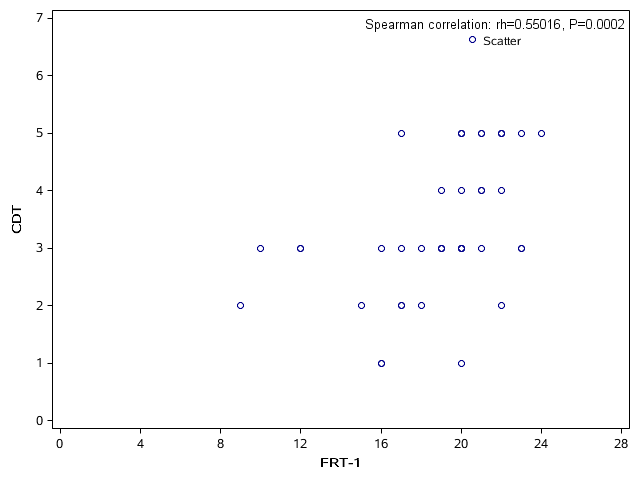
 **Supplementary Figure 13.** Scatter diagram between CDT and FRT-1


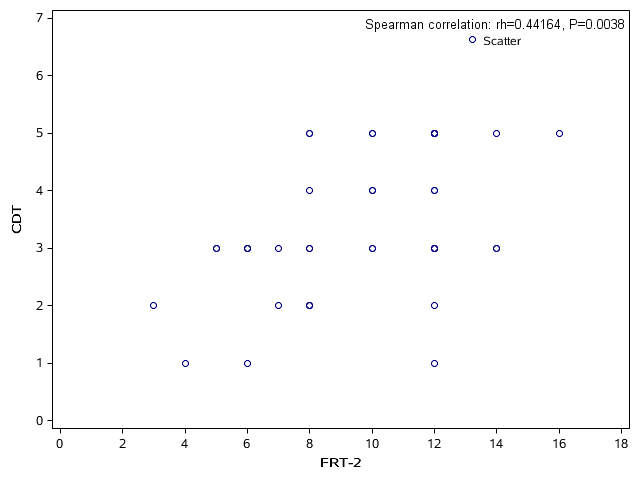
 **Supplementary Figure 14.** Scatter diagram between CDT and FRT-2


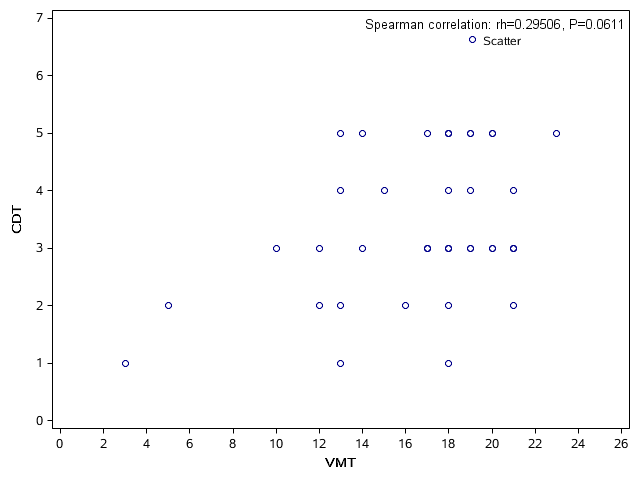
 **Supplementary Figure 15.** Scatter diagram between CDT and VMT


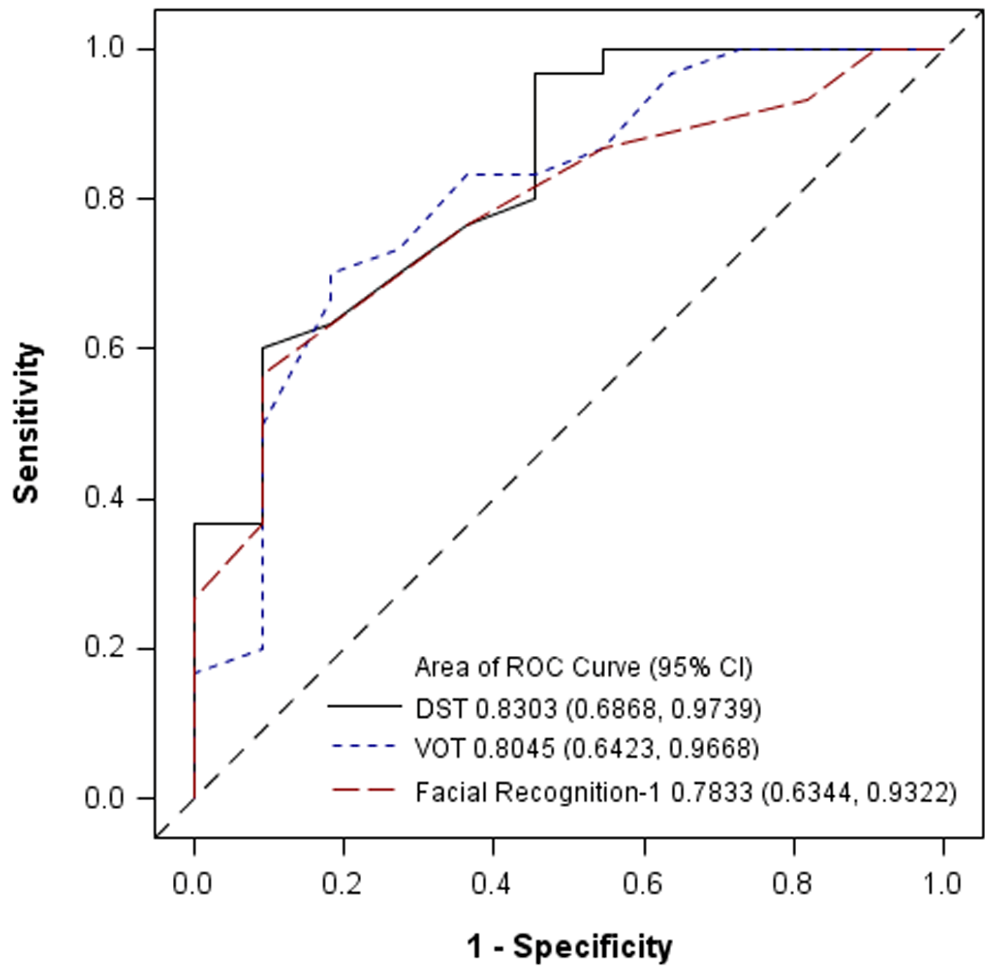
 **Figure 16.** ROC curves of DST, VOT, and FRT-1 for visual-spatial disorder
